# Analysis of coding variants in the betacellulin gene in type 2 diabetes and insulin secretion in African American subjects

**DOI:** 10.1186/1471-2350-7-62

**Published:** 2006-07-25

**Authors:** Steven C Elbein, Xiaoqin Wang, Mohammad A Karim, Winston S Chu, Kristi D Silver

**Affiliations:** 1 Endocrinology Section, Medical Service, Central Arkansas Veterans Healthcare System, Little Rock, AR 72205, USA; 2 Division of Endocrinology and Metabolism, Department of Internal Medicine, College of Medicine, University of Arkansas for Medical Sciences, Little Rock, Arkansas 72205, USA; 3 Division of Endocrinology, Diabetes, and Nutrition, Department of Medicine, University of Maryland School of Medicine, Baltimore, MD, USA

## Abstract

**Background:**

Betacellulin is a member of the epidermal growth factor family, expressed at the highest levels predominantly in the pancreas and thought to be involved in islet neogenesis and regeneration. Nonsynonymous coding variants were reported to be associated with type 2 diabetes in African American subjects. We tested the hypotheses that these previously identified variants were associated with type 2 diabetes in African Americans ascertained in Arkansas and that they altered insulin secretion in glucose tolerant African American subjects.

**Methods:**

We typed three variants, exon1 Cys7Gly (C7G), exon 2 Leu44Phe (L44F), and exon 4 Leu124Met (L124M), in 188 control subjects and 364 subjects with type 2 diabetes. We tested for altered insulin secretion in 107 subjects who had undergone intravenous glucose tolerance tests to assess insulin sensitivity and insulin secretion.

**Results:**

No variant was associated with type 2 diabetes, and no variant altered insulin secretion or insulin sensitivity. However, an effect on lipids was observed for all 3 variants, and variant L124M was associated with obesity measures.

**Conclusion:**

We were unable to confirm a role for nonsynonymous variants of betacellulin in the propensity to type 2 diabetes or to impaired insulin secretion.

## Background

Type 2 diabetes (T2DM) has a substantial genetic component, but identification of susceptibility genes has been limited by the large number of loci and small effect size. Defective insulin action is widely accepted as one element in the progression of metabolic syndrome to impaired glucose tolerance and T2DM, but increasing data support a role for early β-cell dysfunction in the pathogenesis of type 2 diabetes [1-3]. Impaired β-cell function predicts future diabetes [4], and work from our laboratory [5] and others [6,7] suggest that the ability of pancreatic β-cell to compensate for prevailing insulin sensitivity is highly heritable. Mutations in β-cell transcription factors suggest that control of both β-cell mass and insulin secretion may play a role in the genetic susceptibility to β-cell failure and T2DM [8].

Betacellulin (BTC) is one of the factors potentially controlling β-cell growth. Although the BTC gene is located on chromosome 4q13-q21, which is not a replicated region of linkage to T2DM, BTC is nonetheless a strong candidate for T2DM. BTC, which was isolated from insulinoma cells, is highly expressed in pancreas and intestine [9]. BTC belongs to the epidermal growth factor (EGF) family and appears to act through the EGF receptor, although other receptors including ErbB-4 and perhaps a specific BTC receptor have been proposed [10]. Several models suggest that BTC can act to induce neogenesis of β-cells with resultant improvement in glucose homeostasis. Recombinant human BTC improved glucose tolerance and increased the number of islet-like cell clusters in alloxan-treated mice, suggesting increased islet neogenesis from ductal cells [9]. Activin A and BTC together significantly reduced plasma glucose, increased pancreatic β-cell mass, and increased islet insulin content in streptozotocin treated rats [11]. Rats treated with BTC alone after 90% pancreatectomy also experienced increased β-cell mass, increased islet insulin content, and improved glucose. Finally, BTC induced differentiation of the pancreatic exocrine cell line AR42J into insulin secreting cells [12,13].

Silver et al. demonstrated that BTC was expressed in 9–24 week human fetal pancreas [14]. They established that the human BTC gene contains 6 exons spanning at least 40 kb, of which the first 5 exons are translated. Three nonsynonymous coding variants were identified: Cys7Gly (C7G) in exon 1, Leu44Phe (L44F) in exon 2, and Leu124Met (L124M) in exon 4. The C7G variant showed a 13% lower minor allele frequency in African American cases (frequency 0.32; n = 185) than in controls (frequency 0.45; n = 149; p = 0.0004). Although no other variant was significantly associated with T2DM, and no variant was associated with T2DM in Caucasians, several haplotype combinations, including haplotypes comprising alleles at C7G and L44F, showed significant associations in African American subjects [14].

Recently, Silver and colleagues [15] reported the association of an intron 4 variant of BTC with decreased risk of type 1 diabetes in a case control study of 100 Caucasian cases and 282 Caucasian controls. This study was replicated in 113 informative trios, and provided additional evidence for BTC as a candidate for diabetes risk.

Based on these prior data and the strength of BTC as a candidate for the inherited β-cell defect that contributes to T2DM, we tested the role of these 3 coding variants in African American subjects ascertained in Arkansas in two studies: a case-control study of diabetic individuals with a family history of diabetes and normoglycemic control individuals, and a second study of glucose tolerant individuals who had undergone detailed assessment of insulin sensitivity and insulin secretion. We sought to test the hypothesis that coding defects of the BTC gene predispose to T2DM in African Americans and result in defective insulin secretion in nondiabetic individuals at risk.

## Methods

### Experimental subjects

We genotyped 352 T2DM cases (188 male, 164 female) and 182 controls (91 male, 91 female). Cases were drawn primarily from University of Arkansas for Medical Sciences and Central Arkansas Veterans Healthcare Systems clinics, and were selected for a family history of diabetes in at least one first-degree relative. Control individuals were recruited from the general population, from other ongoing studies, and from spouses of cases; all control individuals had no family history of diabetes in a first degree relative. Control individuals were selected for either a normal 75 g oral glucose tolerance test, or when a glucose tolerance test was not feasible, a random glucose below 5.6 mmol/liter. Demographics of the case-control population are shown in Table 1. Controls were younger (p < 0.001) and leaner (p < 0.001) than cases at the time of testing, but were not different in age at testing from the age of diabetes diagnosis for cases. All subjects provided written informed consent under protocols approved by the University of Arkansas for Medical Sciences Institutional Review Board.

**Table 1:** Demographics of African American Case Control Population

**Trait**	**Controls**	**T2DM**
**Gender (Male/Female)**	95/93	192/172
**Age at visit (years)**	42.6 ± 13.1	54.8 ± 12.6*
**Age of diagnosis**	----	42.6 ± 11.9
**Body Mass Index (kg/m**^2^**)**	30.2 ± 7.2	32.7 ± 7.5*
**Diabetic nephropathy (no nephropathy/nephropathy/unknown)**	----	126/152/88

Available demographic characteristics of case control population. See also Materials and Methods. Individuals with nephropathy had either microalbumin:creatinine ratios over 300, end stage renal disease, or elevated creatinines without another cause. Individuals without nephropathy had 10 years of T2DM with a microalbumin:creatinine ratio under 30. All other individuals, including those with microalbuminuria, were considered unknown. * Significantly different at p < 0.001.

We examined 107 subjects (44 male, 63 female) who had undergone frequently sampled intravenous glucose tolerance tests (FSIGT) under either a tolbutamide-modified protocol (63 subjects) or an insulin-modified protocol (44 subjects). Subjects were selected for a range of BMI and family history of T2DM, but all had normal glucose tolerance tests and no prior history of diabetes or gestational diabetes. Characteristics of the study population that underwent FSIGT testing are shown in Table 2. Subjects were in good health and on no medications known to alter insulin sensitivity or secretion. Women were studied in the follicular phase of the menstrual cycle. Blood pressure was measured using the automated Dinamap Pro 100 V2 (GE Healthcare Technologies, Waukesha, Wisconsin), waist and hip circumference were measured at the umbilicus and at greatest diameter, respectively, and height and weight were measured on a wall mounted stadiometer and digital scale, respectively. Percent fat was measured by dual X-ray absortiometry (DEXA) on a Hologic QDR 4500 Elite (Hologic, Inc, Bedford, MA), unless the subject was too large, in which case bioelectrical impedence was measured. For each FSIGT protocol, we obtained 3 baseline samples for glucose and insulin, after which we infused 11.4 g/m^2 ^glucose as D50 over 60 s. Sampling was continued at 2, 3, 4, 5, 6, 8, 10, 12, 14, 16, and 19 min. At 20 min we gave either 125 mg/m^2 ^of tolbutamide (tolbutamide-modified protocol), or because tolbutamide became suddenly unavailable (no Federal Drug Administration approved United States supplier) during the study, we infused insulin at 0.04 U/kg, each over 30 s. Sampling was continued at 22, 23, 24, 25, 27, 30, 40, 50, 70, 90, 100, 120, 150, and 180 min, and subsequently at 30 min intervals to 240 min if the glucose had not returned to baseline. In a subset of 40 individuals, we raised the glucose to 25 mmol/l at the conclusion of the FSIGT protocol, and maintained it for 30 min, at which time a 5 g arginine bolus was administered over 60 s. Insulin was measured at times 0, 2, 3, 4, 6, 8, and 10 min relative to the arginine bolus. Arginine administered at high glucose has been proposed as a surrogate measure of β-cell mass [16].

**Table 2:** Characteristics of African American Subjects Who Underwent Intravenous Glucose Tolerance Testing

**Trait**	**Mean ± standard deviation**
Gender (Male/Female)	44/64
Age (years)	37.2 ± 9.4
Body mass index (kg/m^2^)	30.5 ± 6.4
Waist circumference (cm)	97.6 ± 19.9
Waist:Hip ratio	0.919 ± 0.108
Systolic blood pressure	106.4 ± 10.7
Diastolic blood pressure	63.1 ± 11.4
Protocol (tolbutamide/insulin)	60/30
Percent fat (DEXA)	32.1 ± 11.2
S_I _(× 10^-4 ^min^-1^/μU/ml)	4.95 ± 9.17
Sg (min^-1^)	0.0194 ± 0.082
AIR_g _(pmol/l)	5013 ± 3996
Disposition Index	2590 ± 2608
Fasting glucose (mmol/l)	4.66 ± 0.56
Fasting insulin (pmol/l)	54.3 ± 36.8

### Genotyping of the three betacellulin polymorphisms

We genotyped the 3 coding variants of the BTC gene: C7G (Exon 1;rs28549760), L44F (Exon 2; no rs number), and L124M (Exon 4; rs11938093). We designed primers flanking each of the polymorphisms using Pyrosequencing Assay Design Software 1.0.6 (Biotage, Inc, Uppsula, Sweden). L44F, L124M, and C7G variants were genotyped using Pyrosequencing on a PSQ-96 machine according to the manufacturer's methods (Biotage, Inc). Because of some questions regarding the reliability of the C7G assay, we confirmed the typing in 41 duplicate samples, and also tested all questionable calls using a *Sma1 *restriction enzyme digest that included an internal cutting site, followed by separation on 2.5% agarose gels and scoring from ethidium bromide stained gels. After redesign of the C7G assay, no errors were detected in over 40 repeated samples for each marker. PCR primers and conditions for the each of the SNPs are shown in Table 3. All data were in Hardy Weinberg Equilibrium.

**Table 3:** Conditions and the Primers Sequences

SNP	FORWARD PRIMER 5' to 3'	REVERSE PRIMER 5' to 3'	SEQUENCE PRIMER 5' to 3'	Anneal Temp.
C7G	*CGAAGAAGGAGGGAGACTT	AATGACTTTCCTCCTGGTTTCCA	GAGCTGGCGCCGCTGC	55°C
L44F	TAGACTGTTTCACAATAAGC	*ACAGTGACTGGTACCTTA	GAAGTCCTGAAACTAATGGC	50°C
L124M	CATTGGAGCAAGGTGTGAGA	*CATGTGCAGACACCGATGA	ACAGATTCTGGTGATTTGT	55°C

* Denotes addition of M13 primer sequence (5'-CACGACGTTGTAAAACGAC-3') to the 5' of primer.

### Statistical analysis

Insulin sensitivity (S_I_) was calculated from FSIGT glucose and insulin data using the MinMod Millenium software [17,18]. Insulin secretion was measured as the acute 2 min – 10 min insulin response to the initial glucose bolus (AIR_g_), and disposition index (DI) was calculated as S_I _*AIR_g_, a measure of the β-cell response to insulin sensitivity [19].

Association of the three variants with T2DM were tested primarily for allelic association using either chi-squared or Fisher's Exact test, as implemented in either the 2by2 program [20] or the HaploView 3.2 program [21]. Haplotypes were constructed from 2 and 3 locus combinations using both HaploView 3.2 [21] and Phase 2.1 [22]. Global significance was judged from the default 100 permutations. Impact of the three variants on quantitative traits in glucose tolerant individuals who had undergone FSIGT was tested using mixed effects models, in which ln-transformed BMI and age were covariates and protocol type (tolbutamide or insulin), gender, and genotype were fixed factors. Traits AIR_g_, DI, S_I_, triglycerides, and cholesterol were all ln-transformed to normality prior to analysis. Marginal means were tested for significance and converted back to the linear scale for presentation of results. Previous studies have suggested that although tolbutamide and insulin modified FSIGT are not interchangeable, the results are strongly correlated and thus the statistical method described here will be valid [23]. Because fewer than 5 individuals were homozygous for the uncommon allele for both L124M and L44P variants, we only examined carrier status for the quantitative traits for both variants (for example, L124/L124 vs L124/M124 + M124/M124).

## Results

None of the three variants were associated with T2DM in our population of 352 diabetic and 182 control individuals by allelic association (Table 4), nor when examined in an exploratory analysis by genotype association (p > 0.4; see Table 5). The three variants were not significantly correlated with each other (r^2 ^< 0.05 for all pairs), and no haplotype block was predicted using standard Gabriel definitions [24]. Variants L44F and L124M were in strong disequilibrium by D' (D' = 0.93), whereas C7G was in only modest disequilibrium with L44F (D' = 0.62) and not in linkage disequilibrium with L124M (D' = 0.07). Given no evidence for haplotype blocks with C7G in our data, we chose to examine all possible combinations of 2 or 3 variants (Table 6). No haplotype was associated with T2DM, either when examined individually by HaploView 3.2 or when tested for global differences in the haplotype distribution between cases and controls by Phase 2.1.

**Table 4:** Association of Betacellulin Coding Variants with Type 2 Diabetes in African American Subjects

**Name**	**Variant**	**Position (nucleotide)**	**Frequency**		**P-value**
				
			Cases	Controls	
C7G	T/G	19 (Exon 1)	0.470	0.486	0.65
L44F	C/T	130 (Exon 2)	0.105	0.083	0.28
L124M	T/A	370 (Exon 4)	0.274	0.294	0.52

Frequencies are shown for the minor allele for each SNP. Variant is shown as major/minor allele, and name is likewise shown as common and uncommon amino acid, as originally described.

**Table 5:** Genotypic Counts for Betacellulin Variants

Name	Case Maj/Maj	Case Maj/Min	Case Min/Min	Control Maj/Maj	Control Maj/Min	Control Min/Min
C7G	80	165	101	39	92	44
L44F	276	69	2	150	30	0
L124M	181	146	23	88	78	14

Raw genotype counts for each coding variant; note that counts vary due to some samples not successfully typed for one or more markers. Major alleles are listed first in Table 4.

**Table 6:** Haplotype Analysis of Betacellulin Coding Variants

**Combination**	**Haplotype**	**Case Frequency**	**Control Frequency**
**C7G/L44F/L124M**	TCT	0.334	0.355
	TCA	0.163	0.151
	GCT	0.290	0.271
	GCA	0.129	0.118
	GTT	0.062	0.075
**C7G/L44F**	TC	0.490	0.500
	TT	0.026	0.031
	GC	0.426	0.395
	GT	0.057	0.073
**L44F/L124M**	CT	0.624	0.627
	CA	0.292	0.268
	TT	0.081	0.098
**C7G/L124M**	TT	0.354	0.380
	TA	0.161	0.151
	GT	0.352	0.346
	GA	0.132	0.123

Predicted haplotypes and frequencies in cases and controls are shown from the Phase 2.1 program. Similar results were obtained from HaploView 3.2. Haplotypes with a predicted frequency of over 5% are shown, with the nucleotides as reported in Table 3. No differences approached significance.

We next examined the role of the three BTC coding variants in 107 individuals who had normal glucose tolerance tests and who had undergone detailed assessment of insulin sensitivity (S_I_) and insulin secretion using the FSIGT. Only L124M altered the acute insulin response to glucose (AIR_g_), with carrier status increasing the response from 481 mg/dl among L124 homozygotes to 661 mg/dl in M124 carriers (p = 0.036; 95% CI 390 mg/dl – 593 mg/dl for L124 homozygotes, 531 mg/dl to 822 mg/dl for M124 carriers). No other variant altered the AIR_g_, the ability of the β-cell to compensate for insulin resistance (disposition index, DI = S_I _*AIR_g_), or in 40 individuals, the maximal insulin secretory response to arginine (AIR_max_). Likewise, genotype did not have any main effects on insulin sensitivity, S_I_.

In exploratory analyses, we found evidence for an interaction of L124M with BMI to influence S_I _(p = 0.042). Furthermore, we tested for genotype effects on the additional available traits of blood pressure (systolic and diastolic), obesity (BMI, percent fat, waist:hip ratio), and lipids (triglycerides, total cholesterol, HDL cholesterol) that were not part of our initial hypothesis. We included gender, genotype, and age in obesity models and gender, age, and ln(BMI) in lipid models. We found genotype effects on triglyceride levels (p = 0.007 for C7G and a trend to significance for L44M with p = .059), HDL cholesterol (p = 0.015 for C7G), and total cholesterol (p = 0.034 for L124M). Marginal means for associated traits are shown in Table 7.

**Table 7:** Marginal Means for Traits Associated with Betacellulin Polymorphisms in Non-diabetic Individuals

**Variant**	**Trait**	**Major/Major**	**Major/Minor**	**Minor/Minor**	**p value**
C7G	triglycerides mmol/l	1.343 (1.131, 1.556)	1.025 (0.881, 1.169)	0.865 (0.653, 1.078)	0.007
C7G	HDL cholesterol mmol/l	1.372 (1.229, 1.515)	1.280 (1.183, 1.377)	1.536 (1.392, 1.679)	0.015
L44F	triglycerides mmol/l	0.953 (0.853, 1.066)	1.174 (0.853, 1.066)	---	0.059
L124M	cholesterol mmol/l	4.675 (4.400, 4.951)	4.255^1 ^(3.967, 4.544)	---	0.038

Marginal means are shown after correction for age, gender, and body mass index for each genotype. For C7G, 26 major homozygotes, 54 heterozygotes, and 25 minor homozygotes were successfully typed. For L44P, we identified 60 homozygotes and 21 heterozygotes (no minor homozygotes observed). For L124M, we identified 57 major homozygotes, 45 heterozygotes, and 4 minor homozygous individuals. ^1 ^Data are shown for Major/Minor + Minor/Minor combined. Lipid data are all in mmol/l. We provide means and 95% confidence intervals, converted to the linear scale for skewed variables. P values are for comparison of genotypes shown in the table, taken from the mixed effects model.

## Discussion

Considerable data support heritable defects in β-cell function leading to increased diabetes susceptibility, but to date only the glutamine to lysine variant at position 23 in the β-cell potassium channel gene (KCNJ11 E23K) is widely accepted [25]. That variant is uncommon in African Americans. Variants that explain the high prevalence of T2DM in African Americans are largely undiscovered. Betacellulin is a strong candidate to cause β-cell dysfunction and T2DM given its apparent role as a β-cell growth factor, the presence of three nonsynonymous coding variants, and the previous association of the C7G variant with T2DM in African Americans [14]. Of the coding variants examined in this study, C7G in exon 1 is in the signal peptide. The minor allele, G7 in humans, is the wild type for mouse, rat, and cow BTC genes. Hence, this position is not conserved across species, and a functional role for this variant is uncertain. The other two positions are conserved across mouse, rat, and human, and might alter protein structure and function. L124M in the transmembrane domain is a conservative substitution, but L44F could alter tertiary protein structure. However, neither variant was associated with T2DM in the earlier study [14], nor do we find evidence for an association with T2DM. We did observe a modest association of homozygosity for L124 with reduced insulin secretion relative to M124 carrier status, but this association would not be significant after Bonferonni correction. Hence, our data do not support a role for BTC variants in either T2DM susceptibility or insulin secretion. BTC polymorphisms, either when considered individually or in haplotype combinations, were not associated with T2DM. Indeed, we found no trend to significance that would support the results of Silver et al [14]. One limitation of our study is that our controls are relatively young, and despite our attempt to screen for family history and glucose tolerance, some controls may still develop T2DM in the future, which would reduce the power of our analysis. Furthermore, our analysis of insulin secretion was based on only 107 individuals who had undergone detailed phenotypic analysis; hence, power in this group was potentially limiting. Nonetheless, only the common homozygous state for L124M showed a trend to an association with altered insulin secretion.

The frequency of the G7 allele was similar in our control population to that reported by Silver et al. [14] (0.498 in the present study, 0.45 by Silver). However, we found a considerably higher frequency of this allele among African American diabetic subjects than was reported by Silver et al. (0.477 in the present study, 0.32 in the study of Silver et al.). The frequency of the L44F variant was similar in both cases and controls in our two studies, whereas L124M was somewhat more common in the present study in both cases (0.27 in the current study, 0.22 in Silver et al.) and controls (0.29 in the current study, 0.24 in Silver et al.). These differences likely represent chance fluctuations in allele frequencies given the size of the two studies. Based on the allele frequencies reported by Silver et al, we would have 85% power to find an association in our population, or based on the minor allele frequency of 0.32, 70% power to detect an OR of 1.6. However, using minor allele frequencies from our study and an OR of 1.5, our power was 62% for C7G, 65% for L124M, and only 33% for L44F. Hence, we could easily have missed effects at any of these variants of the size of PPARG [26] or KCNJ11 [27] reported in previous studies in Caucasians (OR ≈ 1.2).

Alternatively, the differences may represent discrepant allele frequencies based on geographic differences in African American population structure [28]. Notably, the data from Silver et al. showed marked differences in allele frequency between Caucasian and African American populations for both C7G and L44F variants, but not for the L124M variant. Hence, differences in population stratification between Arkansas and Maryland populations might explain the different results of our studies. We have examined over 40 SNPs in candidate genes not specifically selected for large differences between African American and Caucasian frequencies, and found no significant differences between cases and controls. More recently, we examined 25 markers that show marked differences between Caucasian and African American allele frequencies [29], and similarly found no significant association when tested by permutation (10,000 permutations; p > 0.08). Whereas we cannot completely exclude population stratification without a larger marker set and a larger sample size, these data make this an unlikely explanation for our lack of an association. Alternatively, differences in the ascertainment of our case and control populations in Arkansas and Baltimore might have resulted in different environmental or genetic factors that interact with BTC polymorphisms to increase the risk of T2DM.

We did find a significant effect of C7G on triglycerides and HDL cholesterol. Marginal means support a gene dosage effect. Furthermore, individuals homozygous for the common (C7) allele had the highest triglyceride levels, which is consistent with the association with T2DM found by Silver et al. We found a trend towards an association for L44F and triglycerides, with carrier status for the minor allele showing a trend towards higher triglyceride levels. We also found evidence for an association of reduced cholesterol in carriers of the L124 allele. These findings were not based on *a priori *hypotheses, and considering that we performed 8 analyses for 3 SNPs, none of these findings would reach statistical significance at a nominal p < 0.05 were a Bonferroni correction applied for 24 tests. Furthermore, a biological explanation for these findings is not obvious. BTC is expressed in the gut, but a role in lipid metabolism has not been reported. Hence, we cannot exclude a type II error as the explanation for these findings. This association will need confirmation in other sample sets given these limitations.

## Conclusion

We were unable to find a role for coding variants of the BTC gene in either T2DM risk in African Americans or in altered β-cell function in glucose tolerant, African American individuals. This finding contrasts with the earlier report of an association of the C7G variant with T2DM in African American subjects, but is consistent with the apparently non-critical nature of this residue in the signal peptide and the lack of association observed in Caucasian individuals in the earlier study [14]. Based on an allelic association using the case and control frequencies from Silver et al. and the sample size of the current study, we had 98% power to reject the null hypothesis of no difference in allele frequencies. Unidentified gene-gene or gene-environment interactions, differences in our ascertainment methods, or differences in African American populations in different geographical regions may account for our different findings.

## Abbreviations

AIR_G _– acute insulin response to glucose

BMI: body mass index

BTC – betacellulin

DI – disposition index

S_I _– insulin sensitivity index

T2DM – type 2 diabetes

## Competing interests

The author(s) declare that they have no competing interests.

## Authors' contributions

SCE conceived the study design, oversaw patient ascertainment, performed statistical analyses, and prepared the manuscript. XW performed all typing, designed the assays, and assisted with manuscript preparation. MAK assisted with assay development and design, molecular typing, and manuscript preparation. WSC prepared DNA for the studies, assisted with assay design and genotyping. KDS provided information on the SNPs and on University of Maryland results and assisted in manuscript preparation.

## Pre-publication history

The pre-publication history for this paper can be accessed here:


